# Insights on the genus *Acronymolpus* Samuelson with new synonymies and exclusion of *Stethotes* Baly from the fauna of New Caledonia (Coleoptera, Chrysomelidae, Eumolpinae)

**DOI:** 10.3897/zookeys.720.13582

**Published:** 2017-12-11

**Authors:** Jesús Gómez-Zurita

**Affiliations:** 1 Animal Biodiversity and Evolution, Institute of Evolutionary Biology (CSIC-Universitat Pompeu Fabra), 08003 Barcelona, Spain

**Keywords:** Eumolpinae, New Caledonia, new combinations, new synonyms

## Abstract

In this work, several taxonomic problems affecting the recently erected genus *Acronymolpus* Samuelson, 2015, endemic to New Caledonia, are addressed. Two of the three New Caledonian species described in *Stethotes* Baly are transferred to *Acronymolpus* and their priority is recognized over the names proposed in the revision of this genus. Moreover, different forms of *Acronymolpus* always found in sympatry, one reddish and larger, and the other black and smaller, were each given species status in that revision, but they are recognized here as the females and males, respectively, of the same species. The taxonomic summary of these discoveries is: (i) *A. bertiae* (Jolivet, Verma & Mille, 2007), **comb. n.** = *A. meteorus* Samuelson, 2015, **syn. n.**, and *A. turbo* Samuelson, 2015, **syn. n.**; and (ii) *A. jourdani* (Jolivet, Verma & Mille, 2013), **comb. n.** = *A. gressitti* Samuelson, 2015, **syn. n.**, and *A. joliveti* Samuelson, 2015, **syn. n.** New distribution data and the male genitalia and the spermatheca of the two valid species of *Acronymolpus* are described for the first time with reference to taxonomically important characters. Finally, the last New Caledonian species described in *Stethotes* is recognized here as a member of the endemic genus *Taophila* Heller: *T. mandjeliae* (Jolivet, Verma & Mille, 2010), **comb. n.**

## Introduction

The fauna of Eumolpinae in New Caledonia has received considerable attention in recent years, including the description of several genera and many species (e.g., Jolivet et al. 2007, 2010, 2013), the first molecular phylogenies of this fauna (Papadopoulou et al. 2013; Gómez-Zurita and Cardoso 2014), and some revisionary work (Gómez-Zurita 2011a, 2011b), including the revisions of two endemic genera, *Taophila* Heller and *Acronymolpus* Samuelson, both by the entomologist G. Allan Samuelson (2010, 2015). *Acronymolpus* is a member of the Eumolpini characterized, among other things, by a small but bulky, fusiform body (almost diamond shaped in the larger taxa), long antennae and a very short abdomen, with the first ventrite accommodating very large metacoxae and projecting a long, acute triangular process between them. The genus currently includes four species (Samuelson 2015). Important as this study is in highlighting the singularity of the Eumolpinae in the Western Pacific archipelago with a new endemic genus, it was still preliminary in (i) lacking descriptions of a highly relevant taxonomic character such as the penis, and (ii) not fixing an important taxonomic blunder with implications in Eumolpinae systematics and biogeography. Samuelson (2015) was well aware about the first problem, but considered it a necessary weakness “owing to the rarity of specimens, [whereby] three of the species are left intact and not compromised by dissecting” (Samuelson 2015: 94). Unfortunately, in his revision he did not correct the misinterpretation of previous authors, who described species clearly allied to *Acronymolpus* but placing them in a genus of a different tribe, the Typophorini *Stethotes* Baly.

In this article, the opportunity offered by the availability of new material of *Acronymolpus* from the Museum of Natural History of the University of Wroclaw (MNHW, Wroclaw) and the Hungarian Natural History Museum (HNHM, Budapest) is used to address the abovementioned problems and discuss their taxonomic and biogeographic implications. Because the solution to this taxonomic conundrum involves connections with the genus *Stethotes*, an additional observation and taxonomic act involving a species described in this genus but not related to *Acronymolpus* is presented in this manuscript, definitively excluding the presence of the former genus in New Caledonia.

## Materials and methods

The specific material for each taxon treated in the study, including all available label information, is given under each species treatment. Type material of *Stethotes bertiae* Jolivet, Verma & Mille, 2007 as well as a reassessment of other taxa mentioned in this study confirming the main conclusions presented in this work were kindly informed by G. Allan Samuelson (Bishop Museum, Honolulu, Hawai’i), after I shared these results with him. The specimens were dissected and studied using a Leica M80 stereomicroscope. Genitalia were mounted dry next to the specimen and pictures were taken to aid line drawings using a Leica DFC420 digital camera and stacking with the freely distributed software CombineZP (Alan Hadley, distributed by the author: alan@micropics.org.uk). Beetle anatomic features were described using the standard nomenclature proposed by Lawrence et al. (2010) for exoskeletal parts and Wagner (2007) specifically for the spermathecae.

## Results and discussion

### Reassessment of a repeated geographic and taxonomic pattern

An interesting circumstance affecting *Acronymolpus*, unapproachable by A. Samuelson because of his scant material for study and zeal in avoiding dissection, is the fact that the four species that he described are known from two distant areas only, and each locality has two divergent forms of the genus. This repeated pattern may be suggestive of some kind of general process allowing the coexistence of related species of *Acronymolpus* only if they differ enough in some traits, at least anatomically. One of these forms is larger (3.0–3.3 mm), broader, more convex and reddish, and the other one is smaller (2.4–2.6 mm), more slender, less convex and black. These divergent forms would be, respectively, *A. turbo* Samuelson and *A. meteorus* Samuelson from Col d’Amieu and a nearby locality, and *A. joliveti* Samuelson and *A. gressitti* Samuelson from Mont Panié. The availability for this study of MNHW material from a third locality in the Central Chain (L’Aoupinié) showed the coexistence of the same two forms.

However, the dissection of material from all known localities where *Acronymolpus* is present proved that what could be an interesting case of competitive exclusion or niche partitioning of some kind is nothing but sexual dimorphism. The large reddish specimens are always the females and the black, small specimens are the males of two species, one in Col d’Amieu and one in Mont Panié, respectively. Indeed, knowing that these divergent forms represent sexual dimorphic extremes, and that the characters that were used to distinguish them taxonomically are in fact secondary sexual traits, one can concentrate on the traits that help recognizing them as belonging to the same species. One that is obvious is punctation, which is stronger and deeper on the pronotum and even rugose on the elytra of the species in Col d’Amieu, and finer, distinct in the species in Mont Panié (Samuelson 2015). The sexual dimorphism hypothesis was particularly well grounded on data in the case of *A. meteorus* and *A. turbo*, for which a large number of specimens could be studied (see below), and helped establishing a number of relevant taxonomic acts for the genus *Acronymolpus*.

### Taxonomic findings

#### Acronymolpus bertiae

Taxon classificationAnimaliaColeopteraChrysomelidae

(Jolivet, Verma & Mille, 2007) comb. n.

= Acronymolpus meteorus Samuelson, 2015, **syn. n.**
= Acronymolpus turbo Samuelson, 2015, **syn. n.**

##### Material examined.

**IBE-JGZ**: one male and one female, New Caledonia, Aoupinié, refuge, -21.14890 165.32348, 400 m, 29.xi.2008, leg. M. Wanat, beating rainforest, *Acronymolpus bertiae* (Jolivet, Verma and Mille) J. Gómez-Zurita det. 2017. **HNHM**: (1) one male, New-Caledonie, Col d’Amieu, 19.i.1977, leg. Dr. J. Balogh, *Acronymolpus bertiae* (Jolivet, Verma et Mille) J. Gómez-Zurita det. 2017. **MNHW**: (1) two females, New Caledonia, Col d’Amieu (6.5–7.0 km from gate), 21°35.2'S, 165°46.4'E, 450–470 m, 6.i.2007, leg. M. Wanat & R. Dobosz, night coll.; (2) one male, New Caledonia, Col d’Amieu (3 km from gate), 21°35.1'S, 165°47.8'E, 500 m, 6.i.2007, leg. M. Wanat, *Acronymolpus bertiae* (Jolivet, Verma et Mille) J. Gómez-Zurita det. 2017; (3) one male, New Caledonia, Col d’Amieu (3.0 km to gate), -21.58536 165.79319, 500 m, 16.xi.2008, leg. M. Wanat, *Acronymolpus bertiae* (Jolivet, Verma et Mille) J. Gómez-Zurita det. 2017; (4) one female, New Caledonia, Farino, Parc des Grandes Fougères, Pic Vincent track, -21.60948 165.77459, 600–670 m, 17.xi.2008, leg. M. Wanat; (5) one male and one female, New Caledonia, Farino, Parc des Grandes Fougères, track & forest N of Pic Vincent, -21.59929 165.77519, 670 m, 17.xi.2008, leg. M. Wanat, *Acronymolpus bertiae* (Jolivet, Verma et Mille) J. Gómez-Zurita det. 2017; (6) one male, New Caledonia, Sarramea, trail to Dogny, -21.6229 165.8684, 300–560 m, 9.xi.2010, leg. M. Wanat & R. Ruta, *Acronymolpus bertiae* (Jolivet, Verma et Mille) J. Gómez-Zurita det. 2017; (7) one male and one female, New Caledonia, Aoupinié, road to sawmill, 21°09'S, 165°19'E, 420–530 m, 7.ii.2004, leg. M. Wanat; (8) two males, New Caledonia, Aoupinié, gate to meteo station, 21°11'S, 165°17'E, 900–950 m, 8.ii.2004, leg. M. Wanat; (9) one female, New Caledonia, Aoupinié, Goipin road jct., 21°10.8'S, 165°18.1'E, 730 m, 17.i.2007, night coll., lamp & beating, leg. M. Wanat & R. Dobosz; (10) three males, New Caledonia, Aoupinié, 21°11.0'S, 165°17.5'E, 850–900 m, 18.i.2007, leg. M. Wanat & R. Dobosz; (11) two females, New Caledonia, Aoupinié, 21°11.0'S, 165°17.6'E, 650–800 m, 19.i.2007, leg. M. Wanat; (12) one male, New Caledonia, Aoupinié, -21.17539 165.30952, 700 m, 27.xi.2008, leg. M. Wanat; (13) one female, New Caledonia, Aoupinié, -21.18151 165.30048, 790–830 m, 27.xi.2008, leg. M. Wanat; (14) three males and one female, New Caledonia, Aoupinié, refuge, -21.14890 165.32348, 400 m, 29.xi.2008, leg. M. Wanat, beating rainforest; (15) one male, New Caledonia, Aoupinié, -21.18027 165.30005, 800–830 m, 20.xi.2010, ex *Pycnandra benthami*, leg. M. Wanat & R. Ruta; (16) six males and two females, New Caledonia, Aoupinié, Goipin rd jct to gate, -21.1814 165.2879, 850–900 m [one with: 700–900 m], 20.xi.2010, leg. M. Wanat & R. Ruta; (17) one male and two females, New Caledonia, Aoupinié, meteo station to summit, roadside, -21.1788 165.2786, 950 m, 21.xi.2010, leg. M. Wanat & R. Ruta.

##### Remarks.

Jolivet et al. (2007) described *Stethotes bertiae* based on three specimens collected at Col d’Amieu and compared the new species with *S. minuta* Jacoby, *S. similis* Gressitt and *S. mimica* Gressitt, all endemic from New Guinea (Gressitt 1966; Jolivet et al. 2007). The genus *Stethotes* was proposed with descriptions of nine species from Java, New Guinea, Borneo, and the Moluccas (Baly 1865–1867) and later shown to be particularly species-rich in New Guinea (Gressitt 1966; Medvedev 2009), but also recorded from other areas in the Australasian region, including Fiji and Samoa (Maulik 1929; Bryant and Gressitt 1957). In this geographic context, it seemed reasonable to find the genus in New Caledonia as well. But the finding of *Stethotes bertiae* had implications beyond the discovery of a genus that had not been previously recorded from New Caledonia and is not known from Australia either. *Stethotes* belongs to the tribe Typophorini, a lineage most diverse in the Old World, particularly in South East Asia and in the Western Pacific, and this lineage is also the one thriving in Fiji, with important biogeographic connections with New Caledonia (Keppel et al. 2009). Interestingly, the vast majority of Eumolpinae in New Caledonia belong to the tribe Eumolpini (Gómez-Zurita 2011a; Papadopoulou et al. 2013: note that in these works, the names Colaspoidini and Nodinini were used instead of Eumolpini and Typophorini, respectively). To our knowledge, the only exceptions were *Rhyparida foaensis* (Jolivet, Verma & Mille, 2007) and three species of *Stethotes*, incluiding *S. bertiae* (Gómez-Zurita 2011a).

The original description of *S. bertiae* was very generic, without much useful information on characters that could help in recognizing the correct generic placement of the species, except perhaps the two differentiated arrangements of elytral punctation: confused at basal half and aligned at apical half of elytra. However, the original description included a photograph of the holotype (Jolivet et al. 2007: 91). *Stethotes bertiae* has the size, the characteristic fusiform shape, the long antennae, the strong punctation (aligned at apical half of elytra), and most critically, entire tibiae of *Acronymolpus*, a character clearly showing that the species should not be placed in the Typophorini. Moreover, the revision of *Acronymolpus* included one species, *A. meteorus* Samuelson, also collected in the Col d’Amieu and the nearby Plateau de Dogny and sharing all the (apomorphic) peculiarities of *S. bertiae*. Among these, it is worth mentioning the heavy punctation of pronotum and basal half of elytra, the reddish testaceous coloration of abdominal ventrites, and the finely wrinkled hypomera, referred to as “with heavy isodiametric sculpture” by Samuelson (2015). There is no doubt that Samuelson's species is the same that had been described years earlier by Jolivet et al. (2007), and this claim was recently confirmed by G. Allan Samuelson himself, upon our exchange of opinions, by comparing the type of *S. bertiae* with his own specimens (G. Allan Samuelson, pers. comm.).

**Figure 1.**
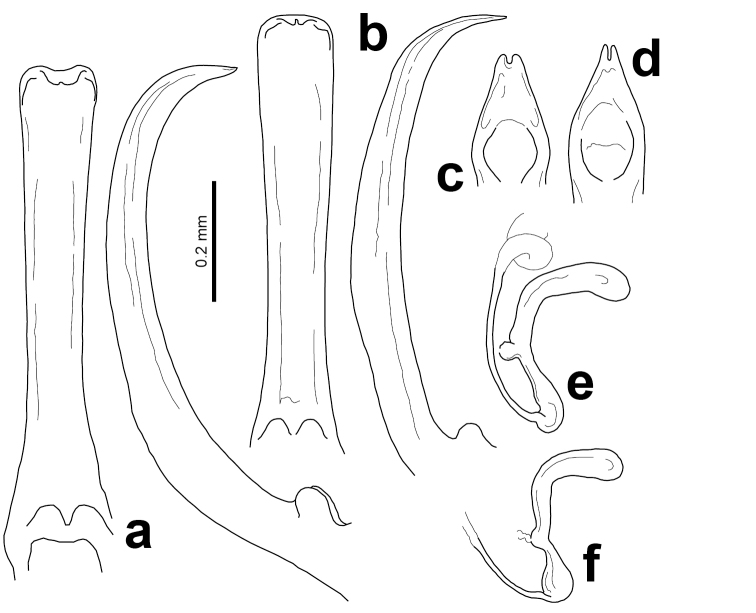
Ventral and lateral views of the penises of **a** *Acronymolpus bertiae* (Jolivet, Verma & Mille) and **b** *A. jourdani* (Jolivet, Verma & Mille). Dorsal view of the distal ends of the penis of **c** *A. bertiae* and **d** *A. jourdani*. Spermathecae of **e** *A. bertiae* and **f** *A. jourdani*.

The original work describing *Stethotes bertiae* included a drawing of the penis, but as it is customary in contributions by the authors of this species, the sexual organ was shown in lateral view, which is of very limited utility for identification purposes (Gómez-Zurita 2011b). In turn, the description of *A. meteorus* lacked any reference to genital structures. The dissection of the new material available for this species showed that they are all males, and male genitalia could be prepared and described focusing on taxonomically relevant characters for the first time (Fig. 1a, c): the penis is narrow and slender, narrower in median part and curved in lateral view, with apex tapering and more strongly bent ventrally, as described by Jolivet et al. (2007); distal end is flattened dorsoventrally, with sides straight and converging to blunt apex with a short median notch. The dissection of the specimens of *A. turbo*, sympatric and syntopic (judging from collection data shown in labels) with *A. meteorus* in every one of the sites where this species has been found, showed that they were all females and, as mentioned above, are interpreted here as conspecific with *A. bertiae*. The spermatheca of the species is described here for the first time (Fig. 1e): spermatheca slender, with nodulus and cornu feebly curved and more or less at right or slightly obtuse angle; cornu thicker than nodulus and blunt at apex; nodulus with bulging insertion of spermathecal gland at middle; spermathecal duct inserted nearly at base of nodulus and bent abruptly.

Based on this complete account, which takes into account geographical but mainly anatomical data and the recognition of sexual dimorphism in the species as described above, three taxonomic acts are necessary. The first is the transfer of *Stethotes bertiae* to the genus *Acronymolpus*, resulting in *Acronymolpus bertiae* (Jolivet, Verma & Mille, 2007), comb. n., and the other two are recognizing that *A. meteorus* (males) and *A. turbo* (females) are junior synonyms of this taxon; thus, *Stethotes bertiae* Jolivet, Verma & Mille, 2007 = *Acronymolpus meteorus* Samuelson, 2015, syn. n. and *Acronymolpus turbo* Samuelson, 2015, syn. n.

#### Acronymolpus jourdani

Taxon classificationAnimaliaColeopteraChrysomelidae

(Jolivet, Verma & Mille, 2013) comb. n.

= Acronymolpus gressitti Samuelson, 2015, **syn. n.**
= Acronymolpus joliveti Samuelson, 2015, **syn. n.**

##### Material examined.

**HNHM**: (1) one male and one female, New-Caledonie, Mont Panié, 7.ii.1977, leg. Dr. J. Balogh, *Acronymolpus jourdani* (Jolivet, Verma et Mille) J. Gómez-Zurita det. 2017. **MNHW**: (1) one female, New Caledonia (N), Mandjélia (summit) 20°23.9'S, 164°31.9'E, 750–780 m, beating, montane rainforest, leg. M. Wanat & R. Dobosz, *Acronymolpus jourdani* (Jolivet, Verma et Mille) J. Gómez-Zurita det. 2017.

##### Remarks.

A few years after the description of *S. bertiae*, Jolivet et al. (2013) described another species of *Stethotes*, *S. jourdani*, very similar to the former but with subtler punctation and from localities further north in the island, in the Massif du Panié, including La Guen, Dawenia, and Wewec (Jolivet et al. 2013). As before, the species does not have any of the characters of the genus *Stethotes*, but these of *Acronymolpus* instead. Interestingly, it fits the description of the second blackish species of *Acronymolpus* described by A. Samuelson a couple of years later, *A. gressitti*, also collected from Mt. Panié. It is worth noting that both descriptions depart in a significant character: the size of the holotypes. The type of *S. jourdani* is reported as 4.0 mm long (Jolivet et al. 2013), while that of *A. gressitti* is 2.6 mm (Samuelson 2015). The measurement given by Jolivet et al. (2013) is far bigger than the largest *Acronymolpus* studied by Samuelson (2015). However, it is important to note that, judging from the figures in the article, the measurements in Jolivet et al. (2013) may not be reliable. *Samuelsonia gomyi* Jolivet et al., 2013 measures 2.5 mm and it shows slightly longer than the holotype of *S. jourdani*, figured alongside and possibly photographed with the same magnification as the former, since both show an identical scale bar of 2.0 mm (Jolivet et al. 2013, figs 7 and 8). Apart from this detail, the two species, *S. jourdani* and *A. gressitti*, match in their characters, as recognized by the author of the latter (G. Allan Samuelson, pers. comm.), including the four basal antennomeres paler, vertex deeply sulcate, smooth hypomera, and pronotal and elytral punctation smaller than in *S. bertiae*.

Moreover, additional material, even if limited, made it possible to draw an analogy with the previous case whereby *A. joliveti* could be recognized as the female of *A. gressitti*. The spermatheca of this species was known due to the description of *A. joliveti*, and the only information available on the male genitalia was a drawing of the penis in lateral view (Jolivet et al. 2013: fig. 1). The spermatheca of *A. jourdani*, figured here from a specimen from Mandjélia, is identical to that figured by Samuelson (2015) from a specimen from Mt. Panié (Fig. 1f). This spermatheca is very similar to that of *A. bertiae*, but shows some relevant differences, including a straight nodulus longer than cornu, conspicuously dilated at base and constricted medially at both ends of bulging insertion of spermathecal gland. Here, the first complete description of the penis of *A. jourdani* is provided and is a very useful character to distinguish this species from *A. bertiae* (Fig. 1b, d). The penis is similar to that of *A. bertiae* with a less pronounced ventral curvature and more gradually curved apex; and the apical end, as seen in dorsal view, has straight sides converging to an acute apex with a deep, narrow median cleft.

As before, three taxonomic acts are required, the first transferring *Stethotes jourdani* to *Acronymolpus*, to propose the new combination *Acronymolpus jourdani* (Jolivet, Verma & Mille, 2013), comb. n., and the second establishing that *A. gressitti* and *A. joliveti* are junior synonyms of this taxon, thus *Stethotes jourdani* Jolivet, Verma & Mille, 2013 = *Acronymolpus gressitti* Samuelson, 2015, syn. n. and *Acronymolpus joliveti* Samuelson, 2015, syn. n.

#### Taophila mandjeliae

Taxon classificationAnimaliaColeopteraChrysomelidae

(Jolivet, Verma & Mille, 2010) comb. n.

##### Remarks.

A third species of Eumolpinae originally ascribed to *Stethotes*, *S. mandjeliae*, was described based on several specimens collected near the summit of Mt. Mandjélia, in the northern part of the Massif du Panié (Jolivet et al. 2010). However, conversely to the other species of *Stethotes* described by the same authors, this species does not belong to the genus *Acronymolpus*. This species unmistakably fits the nominotypical subgenus of *Taophila* instead (Gómez-Zurita and Cardoso 2014), allied to *T. subsericea* Heller, 1916 and *T. corvi* Samuelson, 2010, and easily recognizable by the elongate shape, bicolor antennae, angulated sides of pronotum, which is darker than elytra, marked humeri wider than pronotum, and tapering elytra with partially aligned punctures and stiff hairs, amongst others. It remains to be seen if this species had been previously described by Samuelson (2010), but it may be a valid taxon considering the local area endemism of the Massif du Panié and that no other *Taophila* *s. str.* had been reported in the area. Until the validity of the species is settled, it is appropriate to propose the following transfer: *Taophila mandjeliae* (Jolivet, Verma & Mille, 2010), comb. n.

### Geography of *Acronymolpus* and exclusion of *Stethotes* from New Caledonia

**Figure 2.**
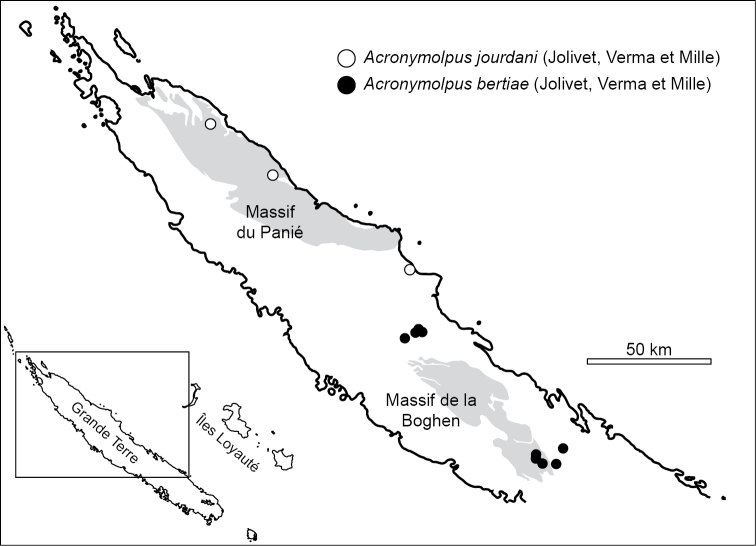
Distribution of *Acronymolpus bertiae* (Jolivet, Verma & Mille) and *A. jourdani* (Jolivet, Verma & Mille) in New Caledonia.

All the localities reported in this study where *S. bertiae* has been confirmed are in the Central Chain, in the northwestern and southeastern edges of the Massif de la Boghen (Fig. 2). Jolivet et al. (2010) reported *Stethotes bertiae* from Mt. Mandjélia, in the northern part of the Massif du Panié, and Jolivet et al. (2013) from a locality in Mt. Panié where they also found *Stethotes jourdani*. It is very likely that these two records relate to the latter species instead. Indeed, the two localities reported here for *A. jourdani* are in the northern part of the Massif du Panié, a separated geographic feature and different area of endemism in New Caledonia relative to the Central Chain and the Massif de la Boghen, where *A. bertiae* occurs (Fig. 2). The holotype of *A. joliveti*, one of the synonyms of *A. jourdani*, is from one locality south from the Massif du Panié, in the valley of one of the rivers discharging in the northern coast of Grande Terre, the Amoa (Samuelson 2015), not so distant from the range of Aoupinié, where the other species, *A. bertiae*, has been found in the current study. However, they are clearly different species based on the analysis of external morphology and their genitalia.

New Caledonia is very rich in species of Eumolpinae of the Eumolpini tribe (Papadopoulou et al. 2013), but the fauna of the archipelago encompassed the Typophorini as well, including one species of *Rhyparida* and three species of *Stethotes* (Eumolpinae: Typophorini) described and reported from Grande Terre (Jolivet et al. 2007, 2010, 2013): *S. bertiae*, *S. jourdani*, and *S. mandjeliae*. This classification had an important implication relative to the biogeography of New Caledonia, namely that the island had been repeatedly colonized by Eumolpinae on, at the very least, three occasions. These would include a minimum of one colonization by Eumolpini, which may have given rise to the high diversity of species in this tribe that we see today in the island (Papadopoulou et al. 2013), once by the Typophorini *Rhyparida*, and at least once by *Stethotes* (Gómez-Zurita 2011a). However, the two most significant findings of this study contradict this interpretation, i.e., the recognition that none of these species supposedly of *Stethotes* belongs to the tribe Typophorini based on the absence of the defining traits of this tribe, and that they belong to two possibly related genera of Eumolpini, *Acronymolpus* in the case of *S. bertiae* and *S. jourdani*, and *Taophila* in the case of *S. mandjeliae*. The fauna of Chrysomelidae of New Caledonia is under intense scrutiny and it is possible that native populations of *Stethotes* may be found in the future. However, at present, the genus must be removed from the faunistic catalogues of the archipelago, which stands out as a center for rich endemic diversity of Eumolpini that may have evolved *in situ* after one or very few colonization events by members of this tribe in the Late Eocene (Papadopoulou et al. 2013).

## Supplementary Material

XML Treatment for Acronymolpus bertiae

XML Treatment for Acronymolpus jourdani

XML Treatment for Taophila mandjeliae
